# Factors Influencing the Acceptability, Acceptance, and Adoption of Conversational Agents in Health Care: Integrative Review

**DOI:** 10.2196/46548

**Published:** 2023-09-26

**Authors:** Maximilian Wutz, Marius Hermes, Vera Winter, Juliane Köberlein-Neu

**Affiliations:** 1 Center for Health Economics and Health Services Research Schumpeter School of Business and Economics University of Wuppertal Wuppertal Germany

**Keywords:** conversational agent, chatbot, acceptability, acceptance, adoption, health care, digital health, artificial intelligence, AI, natural language, mobile phone

## Abstract

**Background:**

Conversational agents (CAs), also known as chatbots, are digital dialog systems that enable people to have a text-based, speech-based, or nonverbal conversation with a computer or another machine based on natural language via an interface. The use of CAs offers new opportunities and various benefits for health care. However, they are not yet ubiquitous in daily practice. Nevertheless, research regarding the implementation of CAs in health care has grown tremendously in recent years.

**Objective:**

This review aims to present a synthesis of the factors that facilitate or hinder the implementation of CAs from the perspectives of patients and health care professionals. Specifically, it focuses on the early implementation outcomes of acceptability, acceptance, and adoption as cornerstones of later implementation success.

**Methods:**

We performed an integrative review. To identify relevant literature, a broad literature search was conducted in June 2021 with no date limits and using all fields in PubMed, Cochrane Library, Web of Science, LIVIVO, and PsycINFO. To keep the review current, another search was conducted in March 2022. To identify as many eligible primary sources as possible, we used a snowballing approach by searching reference lists and conducted a hand search. Factors influencing the acceptability, acceptance, and adoption of CAs in health care were coded through parallel deductive and inductive approaches, which were informed by current technology acceptance and adoption models. Finally, the factors were synthesized in a thematic map.

**Results:**

Overall, 76 studies were included in this review. We identified influencing factors related to 4 core Unified Theory of Acceptance and Use of Technology (UTAUT) and Unified Theory of Acceptance and Use of Technology 2 (UTAUT2) factors (performance expectancy, effort expectancy, facilitating conditions, and hedonic motivation), with most studies underlining the relevance of performance and effort expectancy. To meet the particularities of the health care context, we redefined the UTAUT2 factors social influence, habit, and price value. We identified 6 other influencing factors: perceived risk, trust, anthropomorphism, health issue, working alliance, and user characteristics. Overall, we identified 10 factors influencing acceptability, acceptance, and adoption among health care professionals (performance expectancy, effort expectancy, facilitating conditions, social influence, price value, perceived risk, trust, anthropomorphism, working alliance, and user characteristics) and 13 factors influencing acceptability, acceptance, and adoption among patients (additionally hedonic motivation, habit, and health issue).

**Conclusions:**

This review shows manifold factors influencing the acceptability, acceptance, and adoption of CAs in health care. Knowledge of these factors is fundamental for implementation planning. Therefore, the findings of this review can serve as a basis for future studies to develop appropriate implementation strategies. Furthermore, this review provides an empirical test of current technology acceptance and adoption models and identifies areas where additional research is necessary.

**Trial Registration:**

PROSPERO CRD42022343690; https://www.crd.york.ac.uk/prospero/display_record.php?RecordID=343690

## Introduction

### Background

Health care services worldwide face significant challenges from increasing demand on the one hand and an increasing lack of availability and accessibility on the other hand, accompanied by rising health care costs [1]. The current COVID-19 pandemic has also affected health care delivery and has highlighted the need for alternative approaches that can overcome geographic, temporal, and organizational barriers to providing comprehensive high-quality care [2].

A promising way to overcome these barriers is technological progress, which is driven in particular by increasing digitization and advances in the field of artificial intelligence (AI). One promising technology is conversational agents (CAs), also known as chatbots [3,4]. On the basis of previous definitions, we define CAs as digital dialog systems that enable people to have text-based, speech-based, or nonverbal conversations with a computer or another machine based on natural language. The related concepts and variants of CAs are provided in Multimedia Appendix 1 [5-38].

The use of CAs offers new opportunities and various benefits for health care. Current research points to their ability to improve the accessibility of health care services and medical knowledge and to foster patient-centered care while reducing health care costs. Furthermore, their ability to communicate in multiple languages has been discussed [1,39]. This technology can support health care professionals in their daily work and thus reduce their burden [29]. Numerous studies have demonstrated the effectiveness and efficiency of using CAs in health care, such as in supporting diagnostic decision-making [40] and cognitive behavioral therapy for psychiatric and somatic disorders [41-43]. In this regard, CAs support effective, acceptable, and practical health care comparable with that provided by human physicians [44,45]. Owing to the nonjudgmental nature and impartiality of CAs, studies postulate that the systems may even be better suited than health care professionals to meet the needs of patients in some areas [29].

Achieving acceptability, acceptance, and adoption is challenging for new technologies, as the user’s journey to technology acceptability, acceptance, and adoption is complex and nonlinear [46,47]. The success of an innovation depends on its use by end users, that is, its acceptability, acceptance, and adoption. *Acceptability* is understood as a person’s perception of a technology before its use. *Acceptance*, by contrast, is a person’s perception of a technology after its initial use [46]. *Adoption* refers to a multistage process that explains a person’s choice to use an innovation. It involves a decision-making process that begins with the perception of the technology and ends with the confirmation of the adoption decision or achievement of permanent use [46-48]. The users of the technology must, therefore, be at the center of the digitization process because without including their values and interests in the acceptability, acceptance, and adoption processes, an innovation cannot be successful [46,48-52]. Therefore, it is necessary to determine the factors that affect these processes. Such a broad knowledge base of influencing factors will enable the development of effective strategies for the implementation of new technologies and will serve as a starting point for tailoring new technologies in a user-centric manner, which is crucial for sustainable use [46]. Research regarding the acceptability, acceptance, and adoption of CAs in health care has gained interest tremendously in recent years and has become a significant field.

It is not only private users and patients who are crucial stakeholders within the innovation process of CAs but also staff in health care organizations. In particular, the attitudes and beliefs of staff are crucial for the introduction of CAs to medical institutions because the establishment of new technologies often fails not because of the nature of the systems but because of the employees [50]. One of the most common reasons for the failure of innovations is insufficient knowledge about the acceptability, acceptance, and adoption processes at the time of introduction [53].

Several studies have indicated that despite the benefits of CAs, there is insufficient acceptability, acceptance, and adoption among those who can most benefit from this technology, namely people with health issues [54]. In addition, this technology is usually associated with poor adoption by physicians [39]. To date, several studies have investigated the factors influencing the user acceptability, acceptance, and adoption of CAs in health care. Some factors, such as performance expectations, effort expectations, trust, and facilitating conditions, have already been determined [55-57]. However, a complete overview of the factors influencing the acceptability, acceptance, and adoption of CAs in health care does not yet exist.

### Objectives

This study presents an overview of the facilitating and hindering factors that influence the acceptability, acceptance, and adoption of CAs from the perspectives of patients and health care professionals. Both groups are considered separately to assess whether the influencing factors differ and to derive recommendations for how CAs in the health care system must be designed so that they are used by both patients and health care professionals. Furthermore, CAs can be sustainably integrated into care only if health care professionals are convinced of their benefits and prescribe or recommend them to patients. From the perspective of health care professionals, it is crucial to differentiate between providers’ perception of the use of CAs for patients and their perception of the use of CAs for supporting their own work. On the basis of the identified influencing factors, which were derived from previous technology acceptance and adoption research, a comprehensive thematic map was developed, providing a visualization of the factors that determine the acceptability, acceptance, and adoption of CAs in health care. This up-to-date literature review that shows the factors influencing the acceptability, acceptance, and adoption of health care CAs will enable the design of effective strategies for the implementation and establishment of the technology and user-centered design of the systems, which will lead to sustainable use. Furthermore, the review can serve as a guide for developers, as it shows how the technology should be designed so that it is accepted and adopted by the target group.

These objectives lead to the following research question: what are the factors influencing the acceptability, acceptance, and adoption of CAs in health care from the perspectives of patients and health care professionals?

### Technology Acceptance and Adoption Models

Numerous studies have demonstrated that technology acceptance and adoption models are suitable for investigating the factors influencing technology acceptance and adoption in the health care sector [58,59]. These models attempt to explain the adoption process and use of new technologies and share a basic conceptual framework. This framework explains how individual attitudes affect the intention to use and, ultimately, actual use of new technologies. Researchers from a wide range of disciplines have developed various user acceptance and adoption models for understanding acceptability, acceptance, and adoption from the perspective of individuals or organizations. Table 1 shows the important models and theories of individual acceptance and adoption and their respective determinants.

Whereas previous models could explain between 17% and 53% of an individual’s intention to use a technology, the Unified Theory of Acceptance and Use of Technology (UTAUT) can explain approximately 70% of the variance in an employee’s behavioral intention and up to 50% of the variance in technology use in the organizational context. Moreover, the Unified Theory of Acceptance and Use of Technology 2 (UTAUT2) can explain approximately 74% of the variance in stated behavioral intentions and approximately 53% of the variance in technology use in the consumer context [51,52]. Furthermore, both models have been successfully used in the health care sector on several occasions [55,56,58,59]. Thus, these 2 models serve as the initial models for this review and are described in more detail below.

The UTAUT emerged from a review and synthesis of the 8 most prominent user acceptance models identified in a literature review by Venkatesh et al [51]. The reformulated model includes 4 core determinants, namely performance expectancy, effort expectancy, social influence, and facilitating conditions, which directly determine behavioral intention and use behavior. Unlike previous models, the UTAUT also includes 4 moderators (gender, age, experience, and voluntariness of use) that have a moderating influence on the 4 core determinants [51]. In 2012, Venkatesh et al [52] modified the UTAUT for the consumer technology acceptance and use context to form the UTAUT2. Whereas the UTAUT focuses on predicting the intention to use and actual use of a technology primarily in the organizational context, additional constructs and relationships were identified for the UTAUT2 to predict the intentions to use and actual use of a technology in the consumer context. In particular, the determinants hedonic motivation, price value, and habit were added, and the moderating factor voluntariness of use was removed. Therefore, in the UTAUT2, 7 determinants are moderated by 3 factors [52].

**Table 1:** Models and theories of individual acceptance.

Model	Determinants	Reference, year
TRA^a^	Attitude toward behavior Subjective norm	Fishbein and Ajzen [60], 1975
TAM^b^	Perceived usefulness Perceived ease of use	Davis [49], 1989
TPB^c^	Attitude toward behavior Subjective norm Perceived behavioral control	Ajzen [61], 1991
MPCU^d^	Job fit and complexity Long-term consequences Affect toward use Social factors Facilitating conditions	Thompson et al [62], 1991
C-TAM-TPB^e^	Attitude toward behavior Subjective norm Perceived behavioral control Perceived usefulness	Taylor and Todd [63], 1995
IDT^f^	Relative advantage Ease of use Image and visibility Compatibility Result demonstrability Voluntariness of use	Rogers [48], 1995
MM^g^	Extrinsic motivation Intrinsic motivation	Vallerand [64], 1997
SCT^h^	Outcome expectations—performance Outcome expectations—personal Self-efficacy Affect Anxiety	Compeau et al [65], 1999
TAM 2^i^	Perceived usefulness Perceived ease of use Subjective norm	Venkatesh and Davis [66], 2000
UTAUT^j^	Performance expectancy Effort expectancy Social influence Facilitating conditions	Venkatesh et al [51], 2003
UTAUT2^k^	Performance expectancy Effort expectancy Social influence Facilitating conditions Hedonic motivation Price value Habit	Venkatesh et al [52], 2012

^a^TRA: Theory of Reasoned Action.

^b^TAM: Technology Acceptance Model.

^c^TPB: Theory of Planned Behavior.

^d^MPCU: Model of PC Utilization.

^e^C-TAM-TPB: Combined Technology Acceptance Model and Theory of Planned Behavior.

^f^IDT: Innovation Diffusion Theory.

^g^MM: Motivation Model.

^h^SCT: Social Cognitive Theory.

^i^TAM 2: Technology Acceptance Model 2.

^j^UTAUT: Unified Theory of Acceptance and Use of Technology.

^k^UTAUT2: Unified Theory of Acceptance and Use of Technology 2.

## Methods

### Overview

An integrative review (IR) was chosen to answer the research question. This approach allows the inclusion of studies with diverse methodologies (ie, experimental and nonexperimental research) [67,68] and can precisely represent the state of the current research literature [69]. IRs are the most comprehensive methodological approach to reviews [70] and have many benefits, including identifying gaps in the current research and the need for future studies, evaluating the strength of the scientific evidence, identifying a conceptual or theoretical framework [69], and analyzing methodological issues of a particular topic [71]. Furthermore, the varied sampling frame of IRs in conjunction with the multiplicity of its purpose has the potential to generate a comprehensive understanding of problems related to health care [68].

To ensure methodological rigor, we used Cooper’s [67] 5-stage IR method modified by Whittemore and Knafl [68]. This five-step approach includes (1) problem identification, (2) data collection, (3) data evaluation (quality appraisal), (4) data analysis and interpretation (data extraction), and (5) presentation of results. At the same time, we used the PRISMA (Preferred Reporting Items for Systematic Reviews and Meta-Analyses) checklist as a guide for preparing the IR, which is available in Multimedia Appendix 2 [72].

This review has been registered on PROSPERO (CRD42022343690).

### Information Sources and Search Strategy

To identify the relevant literature for this review, a broad literature search was conducted in June 2021 with no date limits and using all fields in 5 databases (PubMed, Cochrane Library, Web of Science, LIVIVO, and PsycINFO). To keep the IR current, a second search was conducted in March 2022. The search terms were derived from the guiding research question. The following 3 keyword groups were set: “conversational agent,” “acceptability, acceptance, and adoption,” and “influencing factor.” Various synonyms within these keyword groups were generated from the Medical Subject Headings terms of the 5 databases, and further synonyms were derived through a web-based search and from previously published literature discussing CAs. Finally, we used an extensive list of 43 search terms (Multimedia Appendix 3). The search strategy was cross-checked with the Guideline Statement for Electronic Search Strategies [73]. In addition, a preliminary search was conducted in each database to ensure the appropriateness and relevance of the adopted keywords because different digital databases use search engines with different requirements.

### Eligibility Criteria

The PRISMA selection process was used to review publications for inclusion [74]. All studies were assessed against a set of predetermined inclusion and exclusion criteria, which were defined and guided by the research question and purpose of the IR. Studies were included in the IR if they met the following criteria: (1) the language was English or German; (2) the papers were primary studies (3) published until December 31, 2021, and (4) described the acceptability, acceptance, and adoption of CAs and their influencing factors (5) in health care; and (6) the studies adopted a quantitative, qualitative, or mixed methods design (Table 2).

**Table 2:** Inclusion and exclusion criteria.

Characteristics	Included	Excluded
Language	English and German	All other languages
Article type	Published primary study	All article types other than primary studies (eg, reviews) and unpublished primary studies
Year of publication	Inception of the database to December 31, 2021	N/A^a^
Context	Studies that describe the acceptability, acceptance, and adoption of CAs^b^ and studies that describe the factors influencing the acceptability, acceptance, and adoption of CAs	N/A
Area	Health care	All other areas
Study design	Quantitative, qualitative, and mixed methods studies	N/A

^a^N/A: not applicable.

^b^CA: conversational agent.

### Selection and Data Collection Process

Screening of studies for inclusion was independently performed by 2 authors (MW and MH) in 2 stages: title and abstract review and full paper review. There were 5 disagreements between the 2 reviewers. The discrepancies were discussed between the authors and resolved through consensus.

### Data Extraction and Outcomes

Data analysis was conducted via the 4-phase process described by Whittemore and Knafl [68]. During the initial phase (data reduction), we extracted the following information from the studies: the perspectives (of patients and health care professionals) on acceptability, acceptance, and adoption; the wording used in terms of acceptability, acceptance, and adoption of the technology; methodology; theory; study area; number of participants; year; country; and the influencing factors. In addition, it was noted whether acceptability, acceptance, or adoption was part of the research question. In the second phase (data display), we converted the extracted data from the individual sources into a table matrix. During the third phase (data comparison), the factors were analyzed in more detail, paraphrased, and assigned to superordinate categories based on the relationships between them and their underlying meaning.

### Synthesis of Results

The influencing factors were coded through parallel deductive and inductive approaches. In the deductive approach, we searched for statements reflecting the factors proposed by the UTAUT or UTAUT2. During the inductive coding, we developed categories that were not included in the UTAUT or UTAUT2. To verify the identification and classification of the influencing factors by the first reviewer (MW), a second independent reviewer (MH) checked the identification and classification of the influential factors in 10% (8/76) of the included studies that were randomly selected. There was only 1 disagreement between the 2 reviewers. The disagreement was resolved after a short conversation, and the identification and assignment of the first author was followed. The final phase (conclusion drawing and verification) comprised interpreting the information derived from the previous stages [68].

### Risk of Bias Assessment

All the retrieved papers were subjected to a quality assessment. The Mixed Methods Assessment Tool version 2018 was used because it is a critical appraisal tool for reviews that include qualitative, quantitative, and mixed methods studies [75].

One researcher (MW) rated all the identified studies, and a second researcher (JK-N) independently rated 10% (8/76) of the identified studies that were randomly selected. For 1 study, the researchers provided slightly different ratings of quality. However, this difference was quickly resolved and was judged to be sufficiently minor to not question the viability of the other ratings.

## Results

### Study Selection

**Figure 1:**
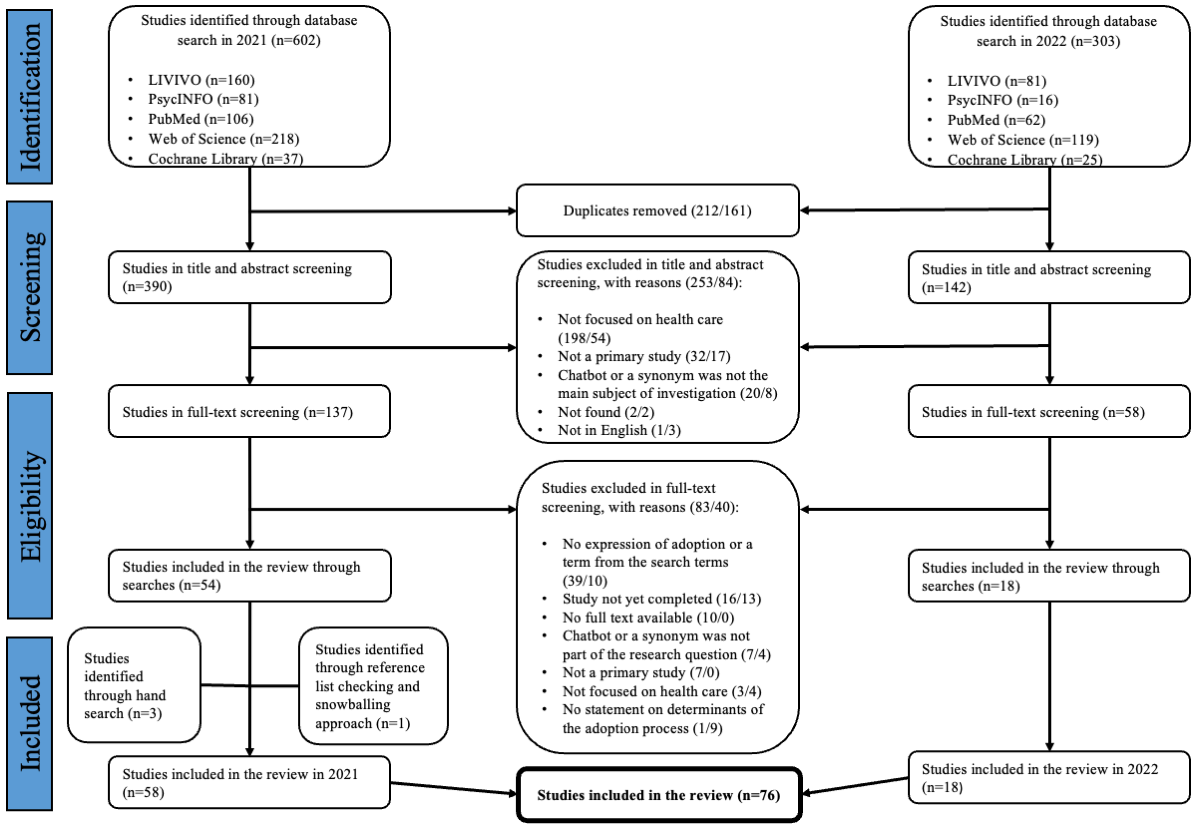
PRISMA (Preferred Reporting Items for Systematic Reviews and Meta-Analyses) flowchart of included studies.

Figure 1 illustrates the flow diagram of the database searches and study screenings.

The first database search yielded 602 studies, and the second database search yielded 303 studies. After duplicates were removed, the titles and abstracts of 532 studies were screened, of which 195 were included in the full-text screening.

Finally, 72 studies were identified through a systematic search. According to Whittemore and Knafl [68], complementary searches are essential for an IR to identify the maximum number of eligible primary sources. Therefore, we used a snowballing approach by searching the reference lists of the eligible studies. In addition, we conducted a hand search. Through the snowballing approach we were able to identify 1 more study and by hand search another 3 studies. A total of 76 studies were included in the review.

### Risk of Bias

The results of the appraisal are available in Multimedia Appendix 4 [2,10,12-29,35-38,42,54-57,76-122]. Overall, the quality of the included studies was high. Because the aim of this IR is to provide a comprehensive account of the factors influencing the acceptability, acceptance, and adoption of CAs in health care, the authors decided to include all studies.

The critical appraisal of the papers revealed a minor risk of bias in 11 (14%) of the 76 publications.

### Study Characteristics

The characteristics of the included studies are summarized in Multimedia Appendix 5 [2,10,12-29,35-38,42,54-57,76-122].

Of the 76 included studies, 69 (91%) exclusively focused on acceptability, acceptance, or adoption among patients, and 3 (4%) focused solely on acceptability, acceptance, or adoption among health care professionals. The remaining 4 (5%) of the 76 studies (the studies by Dupuy et al [22], Kowatsch et al [76], LeRouge et al [26], and Potts et al [77]) explored and described the influencing factors from both the patient and health care professional perspectives.

The 76 included papers were published between 2005 and 2021, with most (n=62, 82%) published from 2019 to 2021. The studies originated from 19 countries. Most studies were conducted in the United States (29/76, 38%) and the United Kingdom (13/76, 17%). The sample sizes of the studies ranged from 4 to 16.519. Of the 76 studies, concerning the study design, 21 (28%) studies had a qualitative design, 17 (22%) had a quantitative nonrandomized design, 15 (20%) had a quantitative randomized controlled design, 14 (18%) had a mixed methods design, and 9 (12%) had a quantitative descriptive design.

Furthermore, the included studies were mostly pilot studies with short intervention periods (mostly between 2 and 4 weeks). Among the 76 studies, there were only 1 (1%) long-term study conducted for >12 months [19] and 2 (3%) studies with a timeframe of >6 months [78,79]. Moreover, the studies were mostly laboratory studies conducted in a controlled environment; only 14 (18%) of the 76 papers used and tested CAs in real-world conditions [25,28,37,56,76,78-86]. Only the studies by Sillice et al [19], Baptista et al [78], and Fan et al [79] were long-term studies under real-world conditions.

The reviewed studies displayed a wide variation in the wording used in relation to the acceptability, acceptance, and adoption of technology. Of the 76 studies, 36 (47%) used the term “acceptability” exclusively, 12 (16%) used the term “acceptance,” and 11 (14%) used the term “adoption.” In addition, of the 76 studies, 8 (11%) studies used both “acceptance” and “adoption congruently,” 7 (9%) used both “acceptance” and “acceptability,” and 1 (1%) used both “acceptability” and “adoption.” Similarly, 1 (1%) study used all 3 terms, (“acceptability,” “acceptance,” and “adoption,”) congruently in their descriptions. By contrast, 3 (4%) studies used none of the terms explicitly but only described the users’ perceptions [35,87,88]. These studies were assigned to the term “adoption” [47]. It was also noted that none of the included studies defined the terms used. Overall, the included studies described a high level of acceptability, acceptance, and adoption of CAs in health care.

Furthermore, only in 10 (13%) of the 76 studies, “acceptability,” “acceptance,” “adoption,” or a synonym was part of the research question or primary research objective [13,21, 22,27,55,56,76,83,89,90]. In 44 (58%) of the 76 studies, “acceptability,” “acceptance,” “adoption,” or a synonym was part of the secondary objectives. Of the 8 studies with an established model to measure the acceptability, acceptance, and adoption of health CAs, 6 (75%) referred to the Technology Acceptance Model [10,86,90-93], and 2 (25%) referred to the UTAUT2 [55,56].

The CAs of the included studies targeted various health domains, as shown in Textbox 1. The textbox shows which health domain was supported by a CA and where it was used within a common medical care pathway. The most CAs dealt with mental health issues and covered the complete care path.

**Health domains targeted by the studied conversational agents (CAs).**

**Prevention**

- Mental health [23,88]
- Family health history [10,20,94]
- Pregnancy care [88,95]
- Health adviser and promoter [81,91]
- Vaccination [96]
- Sexual health advice [57,97]
- Health care for children [98]
- Healthy lifestyle behavior [28,99]
- Exercise and sun protection [19]
- Tuberculosis [92]
- Physical activity [100]
- Cancer [90]
- Diabetes [101]

**Diagnostic**

- Self-diagnosis [55,79]
- Mental health [36]
- COVID-19 [102]

**Treatment**

- Mental health [16,21,24,42,56,77,80,84,103-109]
- Pregnancy care [110]
- Genetic counseling [111]
- Chronic pain [82]
- Diabetes [17,78]
- Sleeping concerns [25]
- Heart disease [18,83,112]
- HIV [14]
- Adiposities [26]
- Smoking cessation [113]
- Substance misuse [93,114]
- Sickle cell disease [115]
- Drug information and risk minimization measures by physicians [116]

**Rehabilitation**

- Physical therapy [76]
- Mental health [38,88,117]
- Cancer [117,118]
- Pregnancy care [88]

**Care**

- Dementia [37]
- Home care [22]
- Care of older people [13,15,27,85-87,119,120]

**General**

- General use of CAs in health care [29,35,54,89,121]
- General use of CAs in relation to COVID-19 [2]

### Influencing Factors

#### Overview

Among the 76 papers, the 73 (96%) papers dealing with acceptability, acceptance, and adoption among patients included 354 mentions of 13 distinct influencing factors. The 7 (9%) of the 73 studies dealing with the acceptability, acceptance, and adoption among health care professionals referred to different health care professional groups. In addition to physicians [26,29,116], the studies investigated the acceptability, acceptance, and adoption of CAs among physiotherapists [76], mental health professionals [77], and (home) care providers [22,85]. In the analysis of the data and description of the results, it was important to distinguish between the perception of health care professionals of the use of CAs for patients and their perception of the use of CAs to support their daily work.

**Figure 2:**
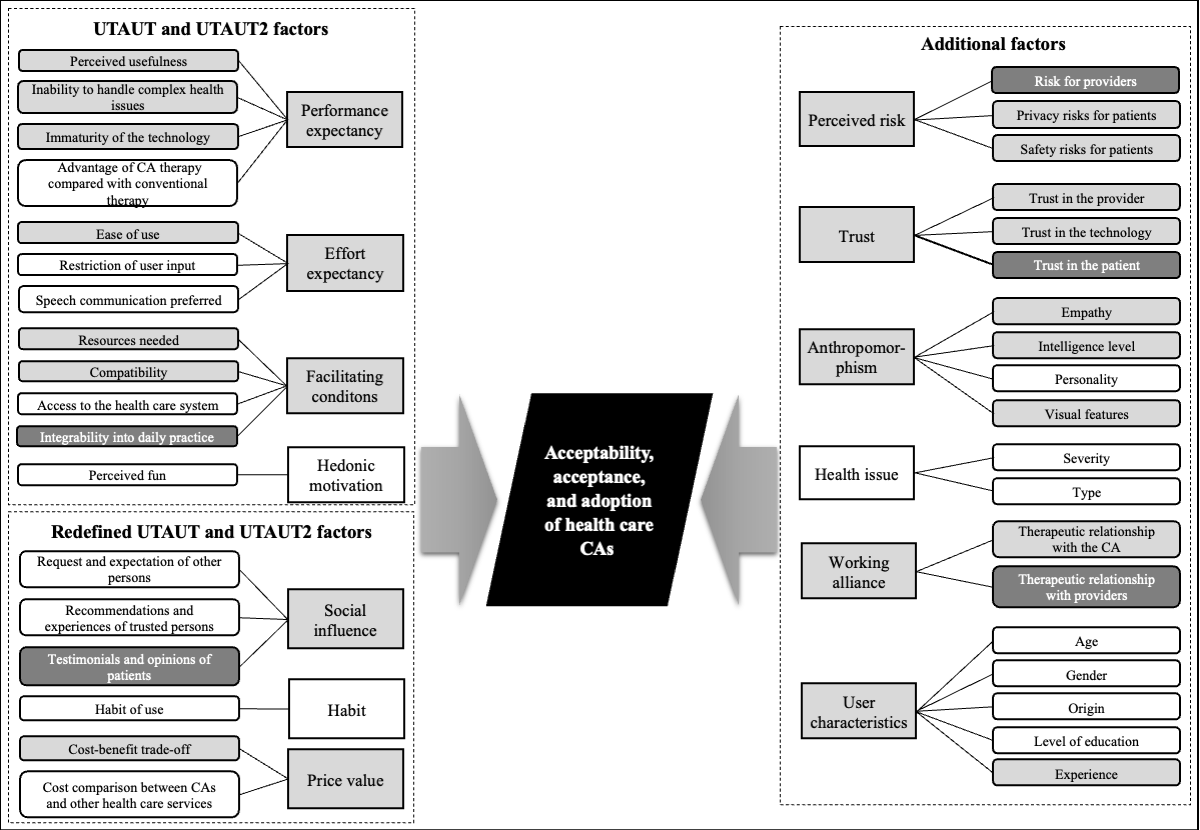
Thematic map of the factors that influence the acceptability, acceptance, and adoption of CAs among patients and health care professionals. CA: conversational agent; UTAUT: Unified Theory of Acceptance and Use of Technology; UTAUT2: Unified Theory of Acceptance and Use of Technology 2.

Figure 2 summarizes the factors, along with their subthemes, that explain the acceptability, acceptance, and adoption of CAs among patients and health care professionals. For a clearer presentation of the influencing factors, different shades of gray are used in the thematic map (influencing factors and subthemes mentioned by both patients and health care professionals are shaded in light gray, those mentioned by patients only are shaded in white, and those mentioned by providers only are shaded in dark gray).

For better comprehensibility within the presentation of results, no distinction was made among the outcomes acceptability, acceptance, and adoption. The original terms from the included primary studies regarding acceptability, acceptance, and adoption can be seen in Multimedia Appendix 6 [2,10,12-28,35-38,42,51,52,54-57,76-84,86-115,117-124] and Multimedia Appendix 7 [22,26,29,51,52,56,76,77,85, 116,123,124] for patients and health care professionals, respectively. In addition, Multimedia Appendices 6 and 7 include a numerical listing of the influencing factors.

#### UTAUT and UTAUT2 Factors

##### Performance Expectancy

*Performance expectancy* refers to the degree to which individuals believe that using a technology will provide them with benefits in performing certain activities [51,52]. According to Venkatesh et al [51], performance expectancy captures the relative advantage [125] and perceived usefulness [49] of the target technology. In 68 (93%) of the 73 studies among patients, performance expectancy was the most frequently identified and researched factor influencing the acceptability, acceptance, and adoption of CAs among patients. A total of 63 (86%) of the 73 studies found that CAs were used by patients when they were perceived as useful and helped them improve their health and quality of life. However, the results from the included studies also suggested that many users rated the performance expectancy of CAs as low because they felt that the technology was not yet sophisticated enough to address complex health issues or detect symptoms of less common health conditions or diseases [54-56,79,97,103,104]. Some studies (2/73, 3%) highlighted that AI at this stage is far too limited and simplistic to be truly effective in many complex health cases [54,56]. Therefore, CAs were more preferred for general questions and interactions with physicians for specific questions [13,27,54,79,97]. The immaturity of the technology was also reflected in the fact that a large number of studies (21/73, 29%) pointed to technical problems with CAs that significantly affected the performance expectancy, acceptability, acceptance, and adoption of the systems. Owing to technical problems, patients did not use the systems or ended the process prematurely [12,13,15,20,21,26,28,37,80,83,90,93,95,96,100,103,104,113,115,118,119]. Moreover, some patients found CAs unhelpful and found talking to a machine disturbing [80,87,97,107,115,117,120].

In addition, some studies (21/73, 29%) reported that patients perceived certain unique advantages of AI-performed therapy over human-performed therapy [13,17,19,21,27,54,56,76-79, 82,90,92-95,97,102,107,111]. Above all, anonymity in the interactions with CAs and the nonjudgmental nature of CAs were strong motivators for their acceptability, acceptance, and adoption and motivated people to use a health CA. Therefore, patients were more willing to share personal, embarrassing, and uncomfortable information with a CA than with a human. Some patients reported that they experienced judgment and blame for their conditions from *real* people [19,21, 42,54,56,77-79,90,93-95,97,102,107,108,115]. Furthermore, patients liked the convenience of a CA-based therapy, which is not possible in a traditional human therapy. They appreciated the ubiquitous availability of CAs and the facts that they have no time pressure in their requests, there is no waiting time, they do not disturb the physicians and waste their time unnecessarily, they can repeat questions or ask uninformed questions, and they can repeat or replay the conversation as often as they want. In addition, patients valued receiving personalized medical treatment or advice through CAs because it is exclusively about them and there are no interruptions from other patients. Moreover, that a CA never makes a patient feel alone and always motivates them to improve their health was perceived as pleasant. Some users felt that well-designed CAs can be more accurate and logical than physicians [12,13,17,19,21,26-28,54,56,76,77,82,88,90,92-95,97,107,111]. Furthermore, patients perceived CAs to be faster, more anonymous, and more informative than information pipelines and search engines. Nevertheless, there were concerns that health CAs could affect the overall quality of health care by replacing experienced professionals [54].

With mentions in all of the 7 included studies on health care professionals, performance expectancy was also the most frequently identified and researched factor influencing the acceptability, acceptance, and adoption of CAs among health care professionals. Health CAs were described by health care professionals as important, useful, and promising [22,26,29,76,77,85,116].

Some studies (5/7, 71%) showed that health care professionals expected patients’ use of CAs to significantly improve health care. By using the systems, patients can better manage their health, access to care can be improved, travel times to medical facilities can be reduced, and unnecessary treatment visits can be avoided. Furthermore, health care professionals anticipated that patients would give more information to the CA owing to the anonymity in the interactions. Significant facilitation and benefits from establishing CAs were observed primarily in the areas of scheduling appointments, finding medical facilities, medication reminders, treatment adherence, providing treatment instructions, and requesting health care. In addition, benefits were also expected in physical therapy. In particular, the freedom of time and space for patients when using a CA and the real-time feedback provided during home exercises were seen as major advantages [26,29,76,77,85].

In addition to the benefits that the introduction of CAs will bring to patients, health care professionals expected that the systems could be of great help in their daily work and would make it much easier. Health care professionals assumed that the use of health CAs would free up time that the providers could then use to provide higher-quality and more individualized care to the remaining patients. The main requirement that health care professionals placed on CAs was that they quickly provide accurate medical information [22,29,77,116].

Among health care professionals, technical problems with CAs had a significant impact on perceived usefulness, acceptability, acceptance, and adoption. However, health care professionals were aware that CAs are still at an experimental stage and, therefore, not yet mature enough to take on more complex tasks. However, some health care workers did not consider CAs to be useful for health care and were skeptical about whether the systems could improve the quality of care and facilitate their daily work [29,76,116].

##### Effort Expectancy

*Effort expectancy* is defined as the degree of ease associated with the use of a technology [51,52]. It can relate to the ease of use for consumers or clients [52] as well as the ease of use for employees or providers [51]. In 82% (n=60) of the 73 studies that addressed the patient perspective, the effort expectancy of CAs was described as a significant factor influencing the acceptability, acceptance, and adoption of CAs. In this regard, it was crucial for patients that the CA responds in a pleasant, light-hearted, user-friendly, and interactive manner; that the interface is simple and easy to use; that the CA is easily accessible; and that the explanations are easy to understand. It was perceived as particularly advantageous that the CA provides all types of health care through 1 device [2,10,12, 13,15-26,28,36,37,42,55,56,76-78,82,86-88,90-101,103-105,107-115,118-122]. However, other patients found CA applications too complicated, the technology too fast or too slow, or the input into a digital system too time consuming [2,20,23, 42,55,79,81,95,96,103-105,110]. Overall, it became apparent that users’ requirements for the usability of CAs vary widely. Some patients wished that each user could personalize (eg, with regard to speed and skills) the CA themselves. CAs that were customizable were highly appreciated by patients [12,19,27,28,76,79,82,93,99,101,109,111,113,118,120]. In addition, the usability of CAs was found to affect the usefulness of the systems [92].

Another aspect of usability concerned the restriction on user input during conversations. In most CA applications, the user can only respond with a list of response options instead of a free-text input. However, patients would like to formulate their answers freely so that they can describe the problems as accurately as possible [15,56,82,97,109,115,120]. In addition, some studies (5/73, 7%) found that patients preferred to talk to the CA rather than chat with the system through text [15,78,87,94,121]. These patients wanted the CA to talk, as interactions with physicians also occur via oral conversations. Moreover, it was pointed out that the patient needs to be able to multitask in a text-based conversation, as questions need to be read and answered simultaneously, and attention must be focused on the CA [87]. In the study by Easton et al [24], the participants were able to participate in the development of the CA and its features and preferred to be able to choose between voice and text communication.

Effort expectancy was mentioned in 4 (57%) of the 7 studies among health care professionals as an important factor for the acceptability, acceptance, and adoption of CAs among health care professionals. Health care professionals wanted an accessible and easy-to-use CA that offers easy-to-read information [22,76,77,116]. CAs were considered easier to use by physicians than the currently available databases for health care professionals [116].

##### Facilitating Conditions

*Facilitating conditions* are defined as an individual’s perception of the resources and support available to execute and use a system [51,52]. Facilitating conditions also represent an important factor for the acceptability, acceptance, and adoption of CAs among patients and were mentioned in 18 (25%) of 73 studies among patients. It was frequently pointed out that it is crucial to have the necessary resources to use a CA, such as a cell phone or computer, and to be able to obtain help from others when needed. Thus, higher acceptability, acceptance, and adoption of CAs in health care have been demonstrated when patients possess such resources and support [2,12,15,19,55,57,92,99,120]. Furthermore, the studies pointed out that a reliable internet connection is usually required to use CAs. Some patients had concerns about the internet connection being interrupted or not having any internet access [26,27,77,92]. Therefore, patients considered it important to continue traditional treatment methods in addition to the CA application, as this approach gives those who do not have reliable internet or smartphone access and those who are reluctant to use CAs the opportunity to receive medical care [111].

The compatibility of the CA with its environment was also important for patients. Compatibility in this context can be defined as the perception that the CA is well integrated into the user’s (health) environment. Especially in the health care context, the compatibility of the CA with the existing health care environment could influence the perception of the usefulness of the technology. Patients wanted a health care CA to be multimodal and accessible through various consumer devices that they already have, such as computers, tablets, cell phones, and televisions. In addition, the system should have the ability to interact with other digital services and home devices, such as calendars, smart home technology, and existing medical devices or applications [21,24,26,28,55,113,120].

Another important factor for patients was the perceived access to the health care system [55,97]. This can be defined as the availability of health care services. Some patients reported that they had quick access to physicians and that this discouraged them from using CAs. By contrast, long distances or the unavailability of health care services or physicians can lead patients to use CAs. Perceived access to the health care system may be limited by local, financial, or institutional factors and largely determines perceptions of the usefulness of CAs [55,97].

For high acceptability, acceptance, and adoption of CAs among health care professionals, the systems should be easy to integrate into daily practice, whether before, during, or after a patient’s treatment. Moreover, the technology should be easily connected and combined with other devices [85,116]. However, a lack of internet access in medical facilities was a clear barrier to the acceptability, acceptance, adoption, and installation of CAs [85].

For the use of CAs by patients, health care professionals considered it crucial for the technology to be multimodal and accessible via multiple devices. A lack of internet access was also seen by health care professionals as a challenge and barrier to the use of CAs. Some suggested embedding the CA in a stand-alone program that does not require constant internet access. Thus, the patient would only need to go on the web at certain intervals to update the system [26,77].

##### Hedonic Motivation

*Hedonic motivation* refers to “the fun or pleasure derived from using a technology” [52]. In 29% (n=21) of the 73 studies among patients, patients wanted CAs to have a self-fulfilling value (instrumental value) for them in addition to health benefits, that is, to be hedonic in nature. Thus, the enjoyment of using health CAs is also a crucial factor influencing their acceptability, acceptance, and adoption. Some studies (3/73, 4%) suggested that a lack of fun makes CAs boring to use and, therefore, decreases their acceptability, acceptance, and adoption [23,76,78].

However, Laumer et al [55] stated that hedonic motivation is not an important factor influencing the acceptability, acceptance, and adoption of CAs in health care. They argued that hedonic motivation is important when a CA serves entertainment purposes but is irrelevant when a CA serves a more serious purpose, such as in the health care domain.

Hedonic motivation was not found to be an influencing factor for acceptability, acceptance, and adoption among health care professionals in the included studies.

##### Social Influence

*Social influence* describes the extent to which an individual perceives that important others (eg, family and friends) believe that the individual should use a particular technology [51,52]. In 11% (n=8) of the 73 studies among patients, social influence was suggested to be an important factor for the acceptability, acceptance, and adoption of CAs among patients. Furthermore, 1 (1%) study found that social influence could affect the performance expectancy of a CA [92]. The results of our analysis showed that the definition of social influence according to Venkatesh et al [51,52] was not sufficient for the application of CAs in the health care sector. Not only was the request or expectation of a certain behavior important but also the recommendation and experience of a person whom the individual trusts [55]. The collected studies showed that patients value the recommendations and experiences of trusted people and would accept and use a CA simply based on testimonials from their social environment [22,26,55,56,92,111].

Social influence was also described by 29% (n=2) of the 7 studies among health care professionals as an important factor for the acceptability, acceptance, and adoption of CAs by health care professionals. Some health care professionals were convinced of the benefits of CAs in health care and would, therefore, recommend the technology to their colleagues. Furthermore, some studies (2/7, 29%) were able to establish a positive correlation between the perceptions of the CA by health care professionals and patients. If a patient perceived a CA as acceptable and useful, this was accompanied by a positive assessment of the CA by health care professionals [22,29].

According to the previous descriptions, the definition of social influence by Venkatesh et al [51,52] must be extended to include recommendations and experiences of trusted persons to fully describe the social influence on the acceptability, acceptance, and adoption of CAs in health care. In terms of the application of CAs in the health sector, social influence should, therefore, be defined as follows: *social influence* refers to the extent to which a person perceives that significant others (eg, family and friends) believe that the person should use a particular technology or to which the person’s perception is influenced by others’ attitudes toward the use of, intention to use, and actual use of the new technology.

##### Price Value

*Price value* is defined as “consumers’ cognitive trade-off between the perceived benefits of the applications and the monetary cost for using them” [52]. Value for money was described in 7 (10%) of the 73 studies among patients as an important factor influencing the acceptability, acceptance, and adoption of CAs among patients. It was found that the price value represents not only the cost-benefit trade-off but also a comparison of the cost of using a CA with the cost of other health services, such as visiting a physician [55,56].

Health care professionals weighed the perceived benefits of CAs against the financial costs for them and patients. Overall, the systems were seen as a cost-effective extension of health care that can improve its quality [29]. Thus, price value also influences health care professionals’ acceptability, acceptance, and adoption of CAs.

According to Laumer et al [55], the comparison between cost and alternative options could be a decisive factor in the acceptability, acceptance, and adoption of CAs, especially in countries with poor insurance coverage or high costs for the use of health care services. In countries with statutory health insurance, such as Germany, patients do not have to pay much for health services. In other countries, such as the United States, patients can incur significant costs depending on their insurance status. Therefore, it is important to compare not only the direct costs of a CA application with the benefits achieved but also the cost-benefit ratio of a CA with that of other health care services [55]. Thus, in terms of the application of CAs in the health sector, the definition of Venkatesh et al [52] should be expanded as follows: *price value* is consumers’ cognitive trade-off between the perceived benefits of the applications and the financial costs of using them as well as the trade-off between the cost of using a CA and the cost of using other health services.

##### Habit

*Habit* refers to “the extent to which people tend to perform behaviors automatically because of learning” [52]. None of the identified studies mentioned habit as defined by Venkatesh et al [52]. However, 1 (1%) of the 73 studies among patients reported that patients would use CAs in health care if they had the habit of using CAs in other areas of their lives [55]. The definition of habit for current health care CA use must, therefore, be expanded to include the extent to which people tend to perform a behavior of interest automatically because they are used to performing a certain action that is close to the behavior of interest [55]. Habit should thus be defined as follows: *habit* describes the extent to which people tend to perform a behavior of interest automatically because of learning and because they are used to performing a certain action that is close to the behavior of interest.

Habit was not found to be an influencing factor for acceptability, acceptance, and adoption among health care professionals in the included studies.

#### Additional Factors

##### Perceived Risk

*Perceived risk* refers to users’ perceived uncertainty of the possible negative consequences of using health CAs [56]. The perceived risk in relation to the acceptability, acceptance, and adoption of CAs among patients was reported in 23 (32%) of 73 studies among patients as an important influencing factor and could be divided into the subtopics of perceived data privacy risk and perceived security risk.

A significant barrier to the acceptability, acceptance, and adoption of health CAs was patients’ concern about potential data privacy risks [2,14-16,21,27,28,54-57,86,97, 107,110,111,118-120]. Users often lacked confidence in CAs’ privacy policies and data-sharing practices and in the ability (or inability) of these systems to maintain confidentiality so that their sensitive health-related information was protected from potential hacking or data leakage [14,54,56]. In particular, the access of other data on the end device (eg, photographs, call logs, or location data) by the CA was viewed critically by patients. In addition, the rate of acceptability, acceptance, and adoption of CAs decreased if they were accessible via third-party services, such as Facebook (Meta Platforms, Inc), as there was a fear that the data would be passed on to third parties [56]. Especially with regard to health issues, data protection was particularly important for users, as the data can be extremely sensitive [14,54-56]. Because automated agent-assisted therapy, unlike conventional therapy, offers the prospect of patient anonymity, users expected their data and identity to be protected [56]. Furthermore, privacy concerns were found to lower the performance expectancy for CAs in addition to acceptability, acceptance, and adoption [55]. To alleviate privacy concerns, patients wanted the security of the CA to be made clearer and the CA to be offered via a trusted tool. In addition, access to data should always be password protected [14,27,111].

Moreover, there were concerns about the risk to user safety and well-being when using CAs in health care. Many patients were unsure about the quality and accuracy of the health information provided by CAs and feared that their use could lead to misdiagnosis. Furthermore, some studies (10/73, 14%) also highlighted criticism about how CAs could put users at risk when used for health issues. Misunderstandings could occur between a CA and its user, who may not be able to accurately describe their health problem or symptoms. In addition, the use of CAs may exacerbate the health problem instead of curing it. Finally, the use of CAs could also lead to increased loneliness and isolation, as it encourages users to seek help from a device rather than from a fellow human [2,21,27,28,54,56,77,88,110]. There were also concerns that patients would be disadvantaged or penalized if they did not use the CA offered [111].

However, in 1 (1%) of the 73 studies among patients, patients had no concerns about data security or an individual security risk when using a CA. There were no privacy concerns, as patients felt that sharing and entering data was commonplace in today’s society. In addition, they felt safe using a CA and, therefore, would not be concerned about being harmed by it [24].

Regarding health care professionals, 5 (71%) of the 7 studies among health care professionals stated that the risk associated with the use of CAs was an important factor in their acceptability, acceptance, and adoption. Their worries could be divided into the subtopics of perceived safety risk for patients, perceived risk for health care professionals, and perceived privacy risk.

Health care professionals feared safety risks from the use of CAs, both for patients and for themselves. They were concerned that CAs could compromise the quality of health care. They were also worried that patients would abuse CAs, incorrectly self-diagnose, and not properly understand the diagnoses displayed. They were also concerned that the systems could indirectly affect the safety and well-being of users by not knowing all the personal factors or not being able to properly clarify issues owing to inaccurate medical information [29]. Furthermore, many health care professionals believed that CAs would play an important role in health care in the future and feared that the systems could replace human workers [29,116]. In addition, there were significant concerns about whether sensitive health-related information was protected from potential hacking or data loss when using CAs [76,85,116].

##### Trust

In 49% (n=36) of the 73 studies among patients, trust was identified as another important factor in the acceptability, acceptance, and adoption of CAs among patients. *Trust* can be defined as “a psychological state comprising the intention to accept vulnerability based upon positive expectations of the intentions or behavior of another” [123]. In terms of the acceptability, acceptance, and adoption of health CAs among patients, trust is not a monolithic concept and should be differentiated into “trust in the provider” and “trust in the technology” [126].

For health CAs, patients’ trust in the provider was a critical factor and played a crucial role in whether the patients would ultimately accept, adopt, and use the CAs [27,55-57,102,107,111].

To have confidence in the technology, patients had to be able to rely on the CA’s capabilities. The reliability, professional competence, and functionality of the system were particularly crucial in this regard. In health care, this means that the CA correctly diagnoses the disease and that the information comes from a credible and evidence-based source. For patients, the comparison between the existing relationship of trust with physicians and the relationship with a health care CA was particularly important. Therefore, it was crucial that patients establish a relationship of trust with a CA similar to that with a physician [13,18,21-23,25,27,35,54-56,77-79,81,82,88, 91,93,94,104,107,110-112,120].

In addition, trust was shown to influence other factors such as perceived risk, effort expectancy, performance expectancy, and hedonic motivation [25,36,55,56,102]. The relationship between the 2 aspects of trust clearly showed that higher trust in the provider also increases initial trust in the CA [55]. Philip et al [36] assumed that credibility is the strongest dimension in terms of patient engagement.

In 5 (71%) of the 7 studies among health care professionals, trust was found to be an important factor for the acceptability, acceptance, and adoption of CAs among health care professionals. Health care professionals trusted neither the technology, assuming that CAs could not correctly assess health problems and situations, nor the patients, with whom they associated the use of CAs with frequent self-diagnosis and lack of understanding of the results delivered [29,77,85,116]. For many health care professionals, mutual trust could only be built through face-to-face encounters. With a CA’s constant monitoring of a patient, they feared a negative impact on the trust relationship between them and the patient [76]. Again, this indicates that trust is not a monolithic concept. With regard to health care professionals, trust should be divided into the categories of “trust in the technology,” “trust in the provider,” and “trust in the patients.”

##### Anthropomorphism

*Anthropomorphism* can be described as the assignment of human-like attributes or traits to nonhuman agents or objects such as robots, computers, or animals. CAs are often attributed human-like characteristics owing to their unique ability to converse in natural language [124]. Anthropomorphism was shown in 67% (n=49) of the 73 studies among patients to be a critical influence on the acceptability, acceptance, and adoption of these systems among patients. According to Nadarzynski et al [54], the lack of human presence is one of the main limitations to using CAs. For health CAs, anthropomorphism can be divided into the subthemes of empathy, intelligence level, personality, and visual features.

For patients, it was important that health care CAs have empathic qualities. In this regard, they wanted CAs to be humorous, caring, friendly, empathetic, warm, honest, supportive, and compassionate. In addition, one of the points they liked most about the technology was that the CA is always there when needed and always listens to them. Many patients would, with increased use, even call the CA a friend [14-17,19,23-26,35,38,42,54,56,77,78,82,87,88,93,94, 103-105,108,109,112,113,115,120]. However, some patients were concerned about a lack of empathy and the CAs’ possible inability to understand emotional issues [2,54,97]. Therefore, they perceived the system as nonemotional, rude, or unsympathetic and imagined the conversation as cold and inhuman [54,56,76,95]. However, it has already been demonstrated that the empathic abilities of health care CAs can be comparable with those of a real person [16,104].

In many cases, CAs were attributed human-like personality traits by users [2,15,24,26,56,77,78,86,87,90,93,99,104, 105,109,120]. A light-hearted, fun, and friendly personality was valued [56,78,93,105]. In addition, an authoritarian personality was not desired in a health care context [35,78]. Similar to how an authoritarian health care professional would be less accepted by patients, if the CA was perceived as an authority figure, the system was less accepted by patients [78]. However, some patients had a negative perception of the CA’s personality. The fact that interacting with a CA feels like interacting with a real person caused them anxiety. Therefore, the systems were perceived by these individuals as creepy, scary, and strange [56,104].

Visual features (appearance), for example, an avatar, were decisive in terms of the acceptability, acceptance, and adoption of a CA application [14,17,19,23,24,26,35,38,76-78, 80,83,87,90,93,94,96,97,103,108,111,120]. However, there should be a match between the appearance of the CA and the expectations of the users. Whereas some patients preferred a serious human appearance to discuss important health issues, others preferred a funny character. Some preferred an avatar of a specific gender or age. Overall, it appeared that patients’ requirements for an avatar varied widely. Therefore, it was suggested that patients should be able to configure the appearance of the CA themselves [13,14,19, 23,24,26,38,76,78,87,90,94,108,111,120]. It was also shown that the appearance of an avatar has a crucial impact on whether the CA appears credible and intelligent [14,90]. Moreover, a visual representation of the system increased the perceived usefulness and enjoyment of the technology [26].

The intelligence level of a CA was reflected in its conversational responsiveness and ability to understand user input. A significant problem with CAs was their lack of intelligibility owing to limited vocabulary, accuracy of speech recognition, or error management of word input or output. In many cases, the inputs were not understood. Systems often needed to be asked more than one question to process the input. In addition, CAs’ responses were reported to be unnatural, impersonal, cold, limited, and repetitive, or arbitrary, scripted responses were given. Because system intelligence was considered important by patients, it has a critical impact on the acceptability, acceptance, and adoption of CAs in health care [12,14,15,17,19-21,23,26,35,37,38,42,54,56,77-81,87,88,90,93, 95-97,100,101,103-106,108,113,120]. Furthermore, 1 (1%) of the 73 studies found that the lack of system intelligence has a negative impact on intention to use, usefulness, and trust [37]. In addition, the perfection of natural communication through congruence between verbal and nonverbal communication was crucial to the acceptability, acceptance, and adoption of the CA. Nonverbal cues, such as facial expressions, gestures, posture, and body movements, had a major impact on guided communication, as many individuals inferred the outcome and social meaning of the conversation from nonverbal behavior. Therefore, a CA should also be able to provide and understand nonverbal cues and respond appropriately [24,78,94,120].

Attribution of human characteristics to a CA was mentioned in 5 (71%) of the 7 studies among health care professionals and is, therefore, also a critical factor for acceptability, acceptance, and adoption among health care professionals. Health care professionals ranked the intelligence of CAs as very important [29,77,85,116]. The prevailing lack of comprehension by CAs was also a severe impediment to the acceptability, acceptance, and adoption of the systems among health care professionals [116]. In addition, health care professionals believed that CAs lack the intelligence and knowledge to accurately assess patients’ health concerns and fully address their needs [29].

Health care professionals expressed great enthusiasm for the use of avatars in health care treatment, as they could serve as a motivator for patients. It was crucial for them that the user can customize and personalize the avatar [26]. For their own use of the systems, health care professionals wanted the CAs to have a neutral and professional appearance that they could customize [116]. Furthermore, health care professionals considered it important for the CA to have empathic properties. However, they believed that mutual empathy could only occur in face-to-face encounters and that CAs are currently unable to understand and represent emotions [26,29].

##### Health Issue

Of the 73 studies among patients, 14 (19%) demonstrated that the acceptability, acceptance, and adoption of CAs in health care were also influenced by the severity and type of the health issue. The severity and type of a disease can be defined as the extent of impairment of physical, mental, and social well-being due to physical dysfunction and the reasons for the physical dysfunction. Mild health problems were found to increase the acceptability, acceptance, and adoption rate of CAs; however, for more serious problems, patients were less willing to use a CA and preferred to be treated or advised by a human [54,57,79,83,89,97,107]. Nevertheless, CAs were perceived as more helpful and credible by patients with more severe diseases than those with less severe diseases [25,80,102]. In addition to the severity of the disease, the type of disease could have a decisive effect on the acceptability, acceptance, and adoption of CAs among patients. In particular, patients who were afraid or embarrassed about their illness or symptoms tended to direct their inquiries to CAs owing to the anonymous and nonjudgmental nature of the interactions [13,54,79,97]. In addition, some studies (3/73, 4%) found that CAs could be a viable treatment method for stigmatized health problems [14,79,89]. However, other studies (2/73, 3%) found no effect of the severity and type of the health issue on the acceptability, acceptance, and adoption of CAs [2,36].

The severity and type of the health issue were not found to be influencing factors for the acceptability, acceptance, and adoption of CAs among health care professionals in the included studies.

##### Working Alliance

The therapeutic relationship that exists between a patient and a physician was also found to be crucial for the acceptability, acceptance, and adoption of CAs among patients in 27% (n=20) of the 73 studies among patients. Therefore, this relationship should also exist between users and the technology [13,15,16,23,26,54,76,78,79,82,84,87,88,94,105,112-114]. The *working alliance* in this context can be defined as a therapeutic relationship between a user and a health CA to jointly achieve the desired (treatment) goal. Establishing a good relationship between a CA and the user was essential to encourage continued use of the technology and an important prerequisite for building a therapeutic alliance that benefits the patient [23]. Moreover, this bond was a motivating factor for patients to continue interacting with the CA [15]. A therapeutic alliance was found to be the result of the empathy, care, and trust that health care professionals demonstrated toward patients [16,54]. To build a patient-CA relationship, recall of past interactions with users and some variability in the systems’ verbal and nonverbal behaviors were critical elements [15]. Patients could only build a relationship with a CA if it was human like. If no relationship could be established with the technology, patients did not value its opinion and would not follow its advice [78].

Of the 7 studies among health care professionals, 2 (29%) also described the therapeutic relationship that exists between a patient and a physician as crucial to the acceptability, acceptance, and adoption of CAs by health care professionals. Health care professionals were concerned that the increasing use of CAs would make patients feel less and insufficiently connected to health care professionals [29]. There was skepticism about whether CAs could help build a strong working alliance between patients and health care professionals. Health care professionals also questioned whether a relationship could be established between a CA and a patient, believing that a working alliance could only be established through face-to-face encounters [76].

##### User Characteristics

Of the 73 studies among patients, 40% (n=29) identified multiple user-related factors influencing the acceptability, acceptance, and adoption of CAs among patients. These included the UTAUT2 factor user experience with the technology [52] and demographic factors such as age, gender, origin, and level of education.

*Experience* is defined as “the passage of time from the initial use of a technology by an individual” [52]. In the beginning, the patient was in an exploratory phase with the CA as a new technology, trying out the functions and not really knowing how to handle the device. After some time, the patient mastered the CA and knew exactly how to handle the device and use it specifically to improve their health. This experience made health care through a CA very efficient [16,19,37,93,104,106,114,121]. Moreover, increasing use made the interaction with the CA more familiar, which affected both the trust relationship and the therapeutic relationship between a patient and a CA [15,16,19,79,106]. Thus, temporal use has a decisive influence on the acceptability, acceptance, and adoption of health care CAs.

Furthermore, our review revealed that the given definition of experience is not sufficient for the use of CAs in health care, as, in addition to the time of use, patients’ experience with health care IT support and CAs in general [2,13,27,55,97], as well as their individual technology knowledge, influenced the acceptability, acceptance, and adoption of CAs. Thus, the systems were less accepted and less widely adopted by individuals with low or moderate IT knowledge [15,24,27,54,57,86,87,92,97,102,111,121]. In addition, 1 (1%) of the 73 studies among patients found that patients who searched the internet more frequently for health information had more fun interacting with CAs and attributed more human-like characteristics to the systems [2]. Experience could also be identified as a user-related factor influencing health care professionals’ acceptability, acceptance, and adoption of CAs in health care. Among health care professionals, acceptability, acceptance, and adoption were influenced by their experience with health care IT support and CAs in general, as well as their individual technology knowledge [85]. Therefore, the definition of experience was extended with regard to the acceptability, acceptance, and adoption of CAs in health care as follows: *experience* is defined as the time that elapses since a person first uses a technology as well as their experience using similar technologies and their resulting individual knowledge.

In addition to experience, the demographic factors age [2,25,35-37,83,84,92,111], gender [35,88], origin [2,83,84,102,114,120], and level of education [2,36,114] influenced the acceptability, acceptance, and adoption of health CAs among patients. It was shown that older age was associated with greater use of CAs among patients and that older patients were more engaged and satisfied with the system than younger patients [2,36,37,83,84]. By contrast, 1 (1%) of the 73 studies among patients showed that patients aged <30 years enjoyed interacting with a CA more than those aged >30 years [2]. Furthermore, male patients perceived CAs to be more useful in the health context than female patients [88]. In addition, patients who were less educated rated CAs as more useful than patients who were well educated [36,114]. It was also showed that Black patients used CAs less than patients of other races with otherwise similar characteristics [83]. Furthermore, people of Asian descent perceived CAs as more useful [84,114].

However, it should be noted that some studies (9/73, 12%) failed to identify any influence of user-related factors on acceptability, acceptance, and adoption among patients [2,17,22,23,25,36,54,86,108].

Demographic factors such as age, gender, origin, and education level were not identified as factors influencing acceptability, acceptance, and adoption among health care professionals in the included studies.

## Discussion

### Principal Findings

The objective of this IR was to identify the factors that influence the acceptability, acceptance, and adoption of CAs among patients and health care professionals. We identified 13 factors that influence the acceptability, acceptance, and adoption of CAs among patients and 10 factors that influence the acceptability, acceptance, and adoption of CAs among health care professionals.

We found that performance expectancy and effort expectancy are the most studied factors influencing the acceptability, acceptance, and adoption of CAs in health care. The findings are consistent with the literature on human-computer interaction (HCI). Perceived ease of use and perceived usefulness are described as key factors in predicting the use of technologies in general and CAs in particular [49,127]. Overall, both health care professionals and patients clearly recognize the benefits of CAs in health care, which has already been shown in a number of studies [41,128]. In addition, studies demonstrated that the health care provided by a CA is comparable with that provided by human physicians [44,45]. Thus, the systems represent a cost-effective alternative to the classic therapy option with the same benefits [39,95].

One of the most interesting findings of the analysis was that, in addition to performance expectancy and effort expectancy, anthropomorphism, trust, perceived risk, and working alliance have been identified as having a decisive influence on the acceptability, acceptance, and adoption of CAs in health care and have not previously been considered in UTAUT or UTAUT2.

In accordance with the literature on the theory of anthropomorphism [124], this IR found that patients attribute human-like characteristics to health CAs and try to interact with them as if the systems were human. HCI research has also found that individuals interact with internet-based agents as if they were humans, even when they know that they are computer programs [129]. In addition, previous work has shown that anthropomorphism has a positive effect in terms of continued use and increased satisfaction with the technology [124]. Moreover, previous work on CAs has already indicated that perceived anthropomorphism can influence CA acceptability, acceptance, and adoption [130].

Nevertheless, it was found that the perceived anthropomorphism could also trigger fear and discomfort in some patients. They perceived the CA as creepy, scary, and strange. In the HCI literature, this is known as the “uncanny valley effect.” The uncanny valley theory states that a technology that appears almost human can evoke negative affective reactions in users [131]. The findings obtained are also consistent with the results of other studies on this topic. Although some studies have reported positive effects of anthropomorphic CAs [124], others have shown that anthropomorphism can lead to frustration, confusion, and even a sense of eeriness [132]. Another criticism of anthropomorphism is that users can be deceived into thinking that they are interacting with a real person instead of a system [16]. Therefore, a CA should always be labeled as a machine.

Furthermore, our results support previous literature on trust by showing that trust is not a monolithic concept but must be differentiated into “trust in the provider” and “trust in the technology” from the patient’s perspective. Whereas trust in the provider refers to patients’ beliefs about the provider’s benevolence, integrity, and competence, trust in the technology refers to patients’ beliefs about the system’s benevolence, functionality, helpfulness, and reliability [126,133]. With regard to health care professionals, it was found that they also have little trust in their patients to use the CA correctly and to interpret the given information correctly. Thus, our results show that trust is represented by the categories “trust in the technology,” “trust in the provider,” and “trust in the patient” from the perspective of the health care professionals. To increase the trustworthiness of CAs, it is suggested in relation to research on AI-driven intelligent systems that responses be presented in a meaningful, understandable, and trustworthy format. In addition, users should be provided with a variety of system-related information, including data on the reliability and performance of the system and the source used for the response output. This should enable users to better understand the information displayed and its origin and then decide whether to trust the technology’s recommendation [134]. It is further suggested that credibility can be demonstrated to users through expert vocabulary and appropriate presentation [133]. Nonmedical studies have also demonstrated that credibility in the form of systems’ functionality, capability, reliability, and benevolence can predict the acceptability, acceptance, and adoption of wearable technologies such as CAs [135]. Furthermore, it was shown that for trust building, the user should have a positive impression of the technology. These impressions are influenced by static and dynamic features. Static features include the appearance of the system, and dynamic features include the verbal and nonverbal behaviors of the system [136]. Moreover, in line with the literature on trust, the results show that building trust in automated systems is a major challenge for developers [137]. Furthermore, it is assumed that patients will use CAs only if they trust them. These explanations show that trust is one of the key factors influencing CA acceptability, acceptance, and adoption [36]. Therefore, it is suggested that trust in health CAs should always be systematically assessed before deployment. However, standardized and validated scales to measure trust are lacking, especially in medicine [138].

Another key barrier to the acceptability, acceptance, and adoption of health CAs is the perceived risk of the technology, stemming from the uncertainty around the protection of personal data and the risk to users’ lives and well-being. Concern about data privacy and the fear of misuse of sensitive information are key barriers to the acceptability, acceptance, and adoption as well as to the widespread use of digital health applications [139]. Studies have shown that privacy concerns can be addressed by the automatic transfer of data from an electronic health record and the regular addition of information by health care professionals. In addition, concerns may be addressed by explaining the measures succinctly and presenting them in layperson’s terms [140].

The literature on digital health applications also frequently discussed whether patient safety is compromised [141] and who is responsible if the CA misdiagnoses someone [4]. The results show that health care professionals fear a safety risk not only for patients but also for themselves. They fear that CAs will play such an important role in the future that they could replace human workers and compromise the quality of health care. We believe that this fear is one of the key barriers to the acceptability, acceptance, and adoption of CAs by health care professionals. In line with the literature, our results clearly show that the development of CAs is still in the early stages, is rudimentary, and thus does not jeopardize jobs [139]. Patients are more willing to share confidential information with a CA than with a health care professional because of its anonymous and nonjudgmental nature of the interactions. However, the preferred use of the systems is for minor illnesses. For more serious conditions, patients prefer to seek advice and treatment from a physician [44,45]. Thus, the use of CAs is purely supportive and does not jeopardize employment. This should be clearly communicated to increase the acceptability, acceptance, and adoption of the technology among health care professionals.

A therapeutic relationship is crucial for the success of a treatment [142]. Such a relationship is the result of empathy, care, and trust and can significantly improve the benefits of a health interaction [143]. Empathy is the most important factor in building a working relationship [144]. We were able to identify the factors of empathy, care, and trust as crucial for the acceptability, acceptance, and adoption of CAs in health care. It has already been demonstrated in some studies that a working alliance can be formed between a CA and a user [4,33,34,43]. For establishing and maintaining a relationship between a patient and a CA, memory of past interactions and variability in verbal and nonverbal responses are crucial elements. This finding is consistent with previous research and shows that, for the correct application of relational behaviors, it is necessary to talk about the past and the future [145] and the time spent apart [146]. Bickmore et al [15] suggested designing health CAs such that interactions are initially relatively distant and professional but gradually become more personal, social, and familiar over time. In addition, systems should have a sense of humor as well as empathy and talk to the user about the present relationship to maintain it [34].

Another crucial barrier to the acceptability, acceptance, and adoption of CAs is their lack of comprehensibility and limited communication capabilities. The literature showed that language skills are a major problem and should be urgently improved [3]. Owing to language limitations, CAs currently use predetermined response options because, unlike free-text entry, they can ensure data validity and accuracy and minimize speech recognition errors. This approach is particularly important in a health-related context, as the multiple-choice input modality avoids potentially dangerous effects of misunderstandings due to ambiguous utterances about medical topics in unrestricted text and speech input. At the same time, it clearly communicates to users how they should respond to the system’s output and ensures that the system can understand and process input with high accuracy. It also enables the CA to be more easily accepted and used by people with different computer and language skills [16,147]. However, our analysis shows that many patients did not want a user input restriction while communicating with systems. Instead of choosing between predefined answers, they would like to be able to answer with a free-text entry to describe their health complaints as precisely as possible.

Furthermore, most patients preferred voice-based communication with a CA over text-based communication. The preferred method of communication of CAs was also discussed controversially in the literature. Even within the definition of CAs, there is no consensus on the preferred mode of communication. The advantages of text-based communication are, for example, that text can be indexed, searched, and translated and that it can be easily corrected or improved after completion. Proponents of acoustic communication are of the opinion that speech is more natural and faster than text. In addition, the use of speech can enhance the perceived personality of a CA. Furthermore, systems that allow acoustic communication can also be used by patients with low or no literacy skills [40,148]. Moreover, we found that nonverbal communication also has a decisive influence on the acceptability, acceptance, and adoption of the systems. Nonverbal cues such as facial expressions, gestures, posture, and body movements have a significant impact on guided communication, as they convey empathy, thereby strengthening the therapeutic alliance and trust relationship between patients and CAs [30,33,34,43]. The 55-38-7 rule proposed by Mehrabian and Ferris [31] shows the importance of nonverbal communication and behavior. Communication can be improved only through a combination of verbal and nonverbal behaviors [30]. We believe that all types of communication will be important in health care in the future. Whether written or oral communication is advantageous will depend on the situation in which the CA is used. For example, whereas an oral dialog with a CA may be easier for a human who is severely injured or paraplegic or a human who is illiterate, a written conversation may be beneficial for a prescription transfer or a patient with speech impairment.

One of the main criticisms of CAs in the literature is that they would not be able to develop empathy, recognize users’ emotional states, or tailor their responses to them. A lack of empathy can affect the use of CAs in the health care sector [143]. To increase the acceptability, acceptance, and adoption, as well as effectiveness, of CAs among patients, it is, therefore, important that the systems have the same interpersonal and social characteristics as health care professionals. In addition, empathic responses help create a trusting relationship between the technology and the user, which guarantees continuous and long-term use of the system and increases the benefits for patients [16]. Consistent with the broader literature, our results show that CAs can be empathic [16,33,43]. Some studies even showed that the empathic abilities of CAs can be compared with those of a real person [149]. In health care, empathy as part of anthropomorphism is critical for the success of CAs [38]. It was found that the visualization of a CA in the form of an avatar makes it more credible, comfortable, sympathetic, and useful than a CA without an avatar [33].

The COVID-19 pandemic has had a significant impact on normal health care delivery and has demonstrated the urgent need for alternative approaches that can overcome geographic, temporal, and organizational barriers. The pandemic resulted in limited access to outpatient clinics, and the high rate of infection posed significant challenges to medical facilities, which affected the delivery of health services [2,110]. This situation has clearly demonstrated that the short-term unavailability of health services can occur even when rapid access to services is basically guaranteed. In this regard, technological systems such as CAs are a good alternative for the continued provision of quality care. It has been shown that CAs can improve and facilitate access to health care [150]. In addition, there are concerns about what happens once the internet connection is lost or individuals do not have the necessary resources such as a smartphone or internet access [57]. Services that can be accessed only through technology may lead to a digital divide and inequity in health care. This would limit access to health services, potentially for the very people who need the services most. For example, digital searching for health information is uncommon among older adults and other underserved groups. However, it should be noted that digital technologies expand the availability of health information and resources to many individuals and improve the quality of care [57,151]. Therefore, we propose that health care providers always offer traditional access to health care services alongside technology to provide quality care for everyone and prevent a 2-tier society. Solutions should also be sought to improve the access to digital resources such as the internet that are necessary to access emerging health technologies.

Consistent with Ling et al [152], we found that user-related factors influence the acceptability, acceptance, and adoption of CAs. These include the demographic factors age, gender, origin, and education level as well as the UTAUT2 factor user experience with the technology. Regarding the factors age, gender, and education level, we found different results within the analyzed studies as to whether they influence the acceptability, acceptance, and adoption of health CAs among patients. Other studies on this topic also provided different findings. Although some studies demonstrated the presence of these factors, other studies were unable to do so [135,147]. Furthermore, origin was found to influence the acceptability, acceptance, and adoption of CAs. However, overall, this is an understudied area. Little is known about ethical differences in technology acceptability, acceptance, and adoption. However, in line with previous research, our results show that it is crucial to tailor the technology to the target population and its cultural characteristics [83].

Moreover, it was found that the identified influencing factors influence each other and cannot always be clearly separated. At the same time, our results indicate that the importance of an influencing factor also depends on the purpose of the CA used and the health domain concerned. Multimedia Appendix 8 provides a summary of influencing factors by health domains and health categories (ie, aggregated domains). Whereas in the categories “mental health” and “specific diseases,” anthropomorphism is the most important factor in addition to performance and effort expectancy, credibility and the severity and type of health issue are crucial for CAs as general health advisers and promoters. In the category “pregnancy care and healthcare for children,” by contrast, hedonic motivation is the key influencing variable along with performance and effort expectancy. The importance of the individual determinants based on the purpose of a CA application is, therefore, understandable. However, owing to the small number of studies per health domain and category, this can only be generalized to a limited extent. The mutual influence and not-always-clear separation of the influencing factors as well as their variability and importance depending on the health care domain make the research on the acceptability, acceptance, and adoption of CAs in health care so extensive.

### Strengths and Limitations

As with all studies, this IR has some limitations. One potential limitation is related to the search strategy. It is possible that not all studies on the topic were found despite our comprehensive search strategy, as studies may have discussed the acceptability, acceptance, or adoption of CAs and the influencing factors but used different terms than those we found. In addition, this review included studies published only in English and German, and this approach may have excluded relevant evidence published in other languages. Furthermore, the IR included only primary studies that had already been published, which also excluded relevant studies such as gray literature.

For quality appraisal, we followed the guidelines for rapid reviews [153,154]. A rapid review is a form of knowledge synthesis in which components of the systematic review process are simplified or omitted to produce evidence-based information in a timely manner [155]. As a result, the screening of the studies for the quality assessment of the papers was fully performed by only 1 researcher. A second researcher assessed only 10% (8/76) of the studies. Nevertheless, the expedited process may have introduced biases in quality assessment.

In addition, as the original studies did not consistently define and describe whether they analyzed acceptability, acceptance, or adoption, it was impossible for us to differentiate between these 3 outcomes in our synthesis. Hence, we cannot provide an answer to the question of whether some factors have been researched more frequently or are more influential for one of the outcomes than for the others.

Finally, the findings regarding acceptability, acceptance, and adoption among health care professionals are almost impossible to generalize, as we could only find and evaluate 7 studies on this topic. Owing to the rapid increase in the research literature on this topic, it is possible that new findings already emerged during the preparation and publication of our results and that the review, therefore, no longer reflects the current state of research.

Despite these potential limitations, this IR has several strengths. To our knowledge, this is the first review to provide a comprehensive picture of the acceptability, acceptance, and adoption of CAs and their influencing factors in health care. We described the factors influencing the acceptability, acceptance, and adoption of CAs in health care from the perspectives of patients and health care professionals and created a thematic map that clearly summarizes the findings. Furthermore, the IR follows the same scientific rigor as primary research in that we used Cooper’s [67] 5-step IR method modified by Whittemore and Knafl [68] for its construction. The review was developed, conducted, and reported in accordance with the PRISMA selection process, which allowed us to produce a high-quality review [74]. A total of 5 well-known and frequently used databases in the field of health were searched to retrieve as many studies as possible. The keywords for the search terms used for this purpose were derived from the main research question. Synonyms for the identified keywords were generated using the Medical Subject Headings terms of the 5 databases, a web-based search, and previously published literature on CAs. Freehand searching and forward-backward reference list checks allowed us to identify additional literature missed by the database search and minimize the risk of publication bias. As no restrictions were made with regard to study design, study setting, and country of publication, this review can be considered comprehensive.

### Implication and Future Directions

This IR provides the first comprehensive overview of the acceptability, acceptance, and adoption of CAs in health care and their influencing factors from the perspectives of patients and health care professionals. From the results, it is clear that the acceptability, acceptance, and adoption of CAs from the perspective of health care professionals are significantly underresearched. Other reviews have also found that few studies on CAs have focused on health care professionals [40]. Therefore, future research should urgently explore the acceptability, acceptance, and adoption of CAs among this user group. For this purpose, a survey could be designed based on our theoretical model to confirm the identified influencing factors and determine new ones. Furthermore, health care professionals should also test currently available CAs and provide feedback. In particular, it is crucial to explore the acceptability, acceptance, and adoption of CAs and their influencing factors from physicians’ point of view. Moreover, physicians will only recommend or prescribe CAs if they accept and adopt the technology and are convinced of its benefits. In addition, we demonstrated that health care professionals’ opinions about CAs significantly influence patients. With knowledge about acceptability, acceptance, and adoption among health care professionals, CAs could be sustainably established in health care.

We succeeded in creating a comprehensive thematic map of the factors influencing the acceptability, acceptance, and adoption of CAs. However, the influence of the identified factors on acceptability, acceptance, and adoption as well as the interrelationship among them was not quantitatively validated. Future studies could build on the theoretical model and examine the relative influence of the factors on acceptability, acceptance, and adoption and the dynamics among the factors. Furthermore, the results show that the influence of facilitators and barriers depends on the intended use of a CA and the health domain in which it is used. However, nothing about the strength and importance of the identified factors was mentioned in the analyzed studies. Therefore, future research should also investigate the importance of the individual factors and their interactions with each other for individual areas of care.

We found that CAs have been tested almost exclusively in controlled environments that do not simulate the realistic interactions in clinical practice. It has already been demonstrated that the environment in which the interaction occurs influences technological acceptability, acceptance, and adoption [127,156]. The broader literature also criticized the fact that, to date, most studies have examined the use of CAs in controlled environments rather than in real-world contexts [148,157]. Moreover, most studies into the acceptability, acceptance, and adoption of CAs in health care are short-term studies. However, it has been shown that factors such as habit only develop when the technology is used over a longer period. Therefore, we consider it necessary that CAs be increasingly tested in real environments and over the long term in the future.

Consistent with the current review of Camile et al [46], we noted that, in relation to the technology, researchers attach different meanings to the terms “acceptability,” “acceptance,” and “adoption” and often use them synonymously without referring to established models and definitions from the literature. None of the included studies defined the terms used appropriately or distinguished them from each other. The definitions are often misunderstood, or researchers establish their own definitions. The inconsistent use of the terms “acceptability,” “acceptance,” and “adoption” makes it immensely difficult to compare the results of these studies. For future research, we, therefore, consider it necessary to follow the definitions and established models from the literature on acceptability, acceptance, and adoption, which clearly show the differences among the terms, to achieve consistency, which will allow comparisons across studies and the development of targeted implementation strategies.

### Conclusions

In this review, we identified 13 factors that influence the acceptability, acceptance, and adoption of CAs among patients and 10 factors that influence the acceptability, acceptance, and adoption of CAs among health care professionals. On the basis of the identified influencing factors shown individually for acceptability, acceptance, and adoption, a comprehensive thematic map that explains the acceptability, acceptance, and adoption of CAs in health care was created. Overall, a high level of acceptability, acceptance, and adoption of CAs in health care was observed. This review shows the variety and complexity of influencing factors. Thus, it presents a comprehensive set of factors that can be implemented, improved, or steered to increase the acceptability, acceptance, and adoption of CAs in health care.

To the best of our knowledge, this IR extends the literature by providing the first overview of the research on the acceptability, acceptance, and adoption of CAs in health care. The findings of this review can, therefore, serve as the groundwork for future implementation studies of CAs in health care. Future research should focus on exploring acceptability, acceptance, and adoption from the perspective of health care professionals. Furthermore, it is crucial to test already developed CAs under real conditions and through long-term studies.

## Appendix

Multimedia Appendix 1 Definition, synonyms, and variants of conversational agents.

Multimedia Appendix 2 PRISMA (Preferred Reporting Items for Systematic Reviews and Meta-Analyses) checklist.

Multimedia Appendix 3 Search terms.

Multimedia Appendix 4 Quality appraisal.

Multimedia Appendix 5 Characteristics of the included studies.

Multimedia Appendix 6 Numerical listing of the influencing factors for patients.

Multimedia Appendix 7 Numerical listing of the influencing factors for health care professionals.

Multimedia Appendix 8 Influencing factors by health domains and health categories.

## Abbreviations

AI: artificial intelligence
CA: conversational agent
HCI: human-computer interaction
IR: integrative review
PRISMA: Preferred Reporting Items for Systematic Reviews and Meta-Analyses
UTAUT: Unified Theory of Acceptance and Use of Technology
UTAUT2: Unified Theory of Acceptance and Use of Technology 2

## Data Availability

All data generated or analyzed during this study are included in this published manuscript and its supplementary files.
