# Supplementary material for: Factors Influencing the Acceptability, Acceptance, and Adoption of Conversational Agents in Health Care: Integrative Review
Source: J Med Internet Res. 2023 Sep 26;25:e46548. doi: 10.2196/46548 (PMC10565637; doi:10.2196/46548)
Supplement: Multimedia Appendix 1 [file jmir_v25i1e46548_app1.pdf]

Multimedia Appendix 1: Definition, synonyms and variants of conversational agents

Definition	Digital dialog system that enables people to conduct a conversation with a computer as a conversational partner on the basis of natural language via an interface [5–7].
Synonyms	chatterbot [8,9], conversational interface [7,9], bot [8], conversational system [9], virtual conversational agent [10], multimodal agent [9], smart bot, interactive agent [11], digital assistant [9,11], artificial conversational entity [11], dialogue system [9,12], conversation system, intelligent assistant [9], virtual agent [13], embodied agent [14], relational agent [15–19], relational counselor [20], software agent [21], virtual agent [22–26], intelligent virtual agent [13], intelligent voice assistant [27], intelligent personal assistant [28]
Nature of communication	• Exclusively text-based dialog systems [8,9]<br>• Conversations in the form of both text and speech [5–7,11,29]<br>• The importance of verbal communication (text and speech-based) and non-verbal communication depends on the situation of the CA application. For example, when driving, no instructions should be given via text, and during a lecture, personal notes can only be recorded via microphone with difficulty. In many situations, improved communication necessitates a combination of verbal and non-verbal behaviors [30].<br>• Albert Mehrabian's 55-38-7 rule states that 55% of communicative impact is determined by body language, 38% by tone of voice, and only 7% by content [31].
CAs with a visual representation	• Embodied conversational agent (ECA), or so-called avatars: representation can be abstract, animal-like or human-like and ranges from a simple speech bubble to a moving figure; uses hand gestures, eye gaze, facial cues, and other nonverbal behaviors within a conversation [32].<br>• ECAs can interact in a more personal and human-like way, building trust and a relationship between the system and the users and leading to camaraderie and long-term, continuous use [30,33,34].<br>• Especially in the healthcare sector, the use of and research into ECAs is becoming increasingly common [14,30,34–38].
