# Supplementary material for: Factors Influencing the Acceptability, Acceptance, and Adoption of Conversational Agents in Health Care: Integrative Review
Source: J Med Internet Res. 2023 Sep 26;25:e46548. doi: 10.2196/46548 (PMC10565637; doi:10.2196/46548)
Supplement: Multimedia Appendix 3 [file jmir_v25i1e46548_app3.pdf]

Multimedia Appendix 3: Searchterm

**Search**
variabl* OR determinant* OR parameter* OR barrier* OR driver* OR factor* OR facilitator* OR influence* OR effect* OR impact* OR perception*
**AND**
“technology accept*” OR accept* OR adopt OR adoption OR “behavioral intention*” OR “use behavior” OR “intention to use” OR uptake*
**AND**
chatbot* OR “chat bot*” OR “chat-bot*” OR chatterbot* OR “chatter bot*” OR “conversational agent*” OR “conversational interface*” OR “artificial conversational entitie*” OR “conversational user interface*” OR “conversational system*” OR “dialogue system*” OR “chat agent*” OR “virtual agent*” OR “multimedia agent*” OR “interactive agent*” OR “Intelligent Personal Assistant*” OR “intelligent assistant*” OR “smart bot*” OR “smartbot*” OR “smart speaker*” OR “conversational bot*” OR “embodied agent*” OR “relational agent*” OR “agent based system*”
