# Supplementary material for: Factors Influencing the Acceptability, Acceptance, and Adoption of Conversational Agents in Health Care: Integrative Review
Source: J Med Internet Res. 2023 Sep 26;25:e46548. doi: 10.2196/46548 (PMC10565637; doi:10.2196/46548)
Supplement: Multimedia Appendix 6 [file jmir_v25i1e46548_app6.pdf]

Multimedia Appendix 6: Numerical listing of the influencing factors for patients

Influencing Factor	Definition	Sources Using Acceptance	Sources Using Acceptability	Sources Using Adoption	Numerical Listing
Performance Expectancy	The degree to which individuals believe that using a technology will provide them with benefits in performing certain activities[51,52].	[2,10,13,15,16,22,23,26,36,37,42,55,56,76,79,81,82,86,90–92,94,95,110,115,120,121]	[12,14,17–21,23,24,28,37,38,54,78,82–84,86,90,91,93,94,96–101,103–106,108,109,111–114,117,118,120,122]	[2,13,26,27,55,56,77,79,80,83,86–88,102,107,119,121]	68 (93%)
Effort Expectancy	The degree of ease associated with the use of a technology [51,52].	[2,10,13,15,16,22,23,25,26,36,37,42,55,56,76,79,81,82,86,90–92,94,95,110,115,120,121]	[12,17–21,23,24,28,37,78,82,86,90,91,93,94,96–101,103–105,108,109,111–114,118,120,122]	[2,13,26,55,56,77,79,86–88,107,119,121]	60 (82%)
Facilitating Conditions	An individual's perception of the resources and support available to execute and use a system [51,52].	[2,15,26,55,92,120]	[12,19,21,24,28,57,97,99,111,113,120]	[2,26,27,55,77]	18 (25%)
Hedonic Motivation	"The fun or pleasure derived from using a technology" [52].	[2,15,23,26,56,76,82,86,91,95,110,115,121]	[19,23,28,78,82,86,91,98]	[2,26,56,77,80,86,88,107,121]	21 (29%)
Social Influence	The extent to which a person perceives that significant others (eg, family and friends) believe that the person should use a particular technology, or to which the person's perception is influenced by others' attitudes toward, intention to, and actual use of the new technology.	[2,22,26,55,56]	[17,111]	[2,26,55,56,92]	8 (11%)
Price Value	Consumers' cognitive trade-off between the perceived benefits of	[13,26,55,56,86]	[54,86]	[13,26,27,55,56,86]	7 (10%)

	the applications and the financial costs of using them, as well as the trade-off between the cost of using a CA and the cost of using other health services.				
Habit	The extent to which people tend to perform behaviors automatically because of learning and because they are used to performing a certain action that is close to the behavior of interest.	[55]	N/A	[55]	1 (1%)
Perceived Risk	Users' perceived uncertainty of possible negative consequences of using health CAs [56].	[2,15,16,55,56,86,94,110,120]	[14,21,24,28,54,57,86,94,97,111,118,120]	[2,27,55,56,77,86,88,107,119]	23 (32%)
Trust	"A psychological state comprising the intention to accept vulnerability based upon positive expectations of the intentions or behavior of another" [123].	[13,16,22,23,25,36,55,56,79,81,82,86,90,91,94,110,120],	[14,17,18,21,23,54,57,78,82,86,90,91,93,94,97,104,111,112,120]	[13,27,35,55,56,77,79,86,88,102,107]	36 (49%)
Anthropomorphism	Assigning human-like attributes or traits to non-human agents or objects such as robots, computers, or animals [124].	[2,13,15,16,23,25,26,37,42,56,76,81,82,86,90,94,95,115,120]	[12,14,19–21,23,24,37,38,54,78,82,83,86,90,93,94,96,97,99–101,103–106,108,109,111–113,120]	[2,13,26,35,56,77,80,83,86–88]	49 (67%)
Health Issue	The extent of impairment of physical, mental, and social well-being due to physical dysfunction and the reasons for it.	[25,79]	[14,54,57,83,84,89,97]	[79,80,83,102,107]	14 (19%)
Working Alliance	A therapeutic relationship between a user and a health	[13,15,16,23,26,76,79,82,94]	[23,54,78,82,84,94,105,112–114]	[13,26,79,87,88]	20 (27%)

	CA to jointly achieve the desired (treatment) goal.				
User Characteristics	User Characteristics include demographic factors such as age, gender, origin and level of education, as well as the UTAUT2 factor user experience with the technology.	[2,13,15,16,25,36,37,55,86,92,120,121]	[19,24,37,54,57,83,84,86,93,97,104,106,111,114,120]	[2,13,27,35,55,83,86–88,102,121]	29 (40%)
